# Timing and method of restoration affect fracture resistance of endodontically treated teeth: An in vitro study

**DOI:** 10.1097/MD.0000000000046421

**Published:** 2025-12-19

**Authors:** Yunyu Yuan, Pingping Chen

**Affiliations:** a Department of General Stomatology, Wanshun Outpatient Department of Wuxi Stomatological Hospital, Wuxi, Jiangsu, China.

**Keywords:** endodontically treated teeth, fracture resistance, full crown, occlusal veneer, onlay, restoration timing

## Abstract

Root canal treatment (RCT) often results in significant loss of tooth structure, increasing the risk of tooth fracture under occlusal forces. This study aimed to evaluate the impact of different restoration timings on the fracture resistance of teeth after RCT and to compare the effectiveness of 3 restoration methods: full crown, onlay, and occlusal veneer. Sixty extracted human molars underwent standardized RCT and were randomly assigned to 9 groups (n = 6–7) in a 3 × 3 factorial design: immediate (1 week), early (2–3 weeks) or delayed (4–6 weeks) restoration, each receiving either a zirconia full crown, lithium-disilicate onlay or composite occlusal veneer. After thermomechanical aging (5000 thermal cycles, 50000 chewing cycles, 50 N), specimens were loaded to fracture (1 mm min^−1^). Mean fracture loads declined with delayed restoration: immediate 2 356 ± 413 N, early 2 086 ± 389 N, delayed 1 754 ± 357 N (*P* < .001). Full crowns resisted highest loads, followed by onlays and veneers (*P* < .01); timing × method interaction was significant (*P* = .038). Immediate restoration yielded 85% repairable failures; delay increased non-repairable root fractures to 55% (*P* = .004). Earlier restoration after RCT significantly enhances fracture resistance and clinical prognosis. While full crown restoration offers superior protection, onlay and occlusal veneer are viable alternatives that balance fracture resistance and tooth structure preservation.

## 1. Introduction

Root canal treatment (RCT) is a conventional method for managing dental pulp and periapical diseases.^[1]^ However, this procedure often results in the loss of significant tooth structure, leaving the remaining tooth more susceptible to fracture under occlusal forces.^[2]^ The structural loss caused by caries, access cavity preparation, trauma, and radicular preparation significantly reduces the fracture resistance of endodontically treated teeth.^[3]^ Furthermore, the physical characteristics of the tooth, such as cusps, ridges, and the arching roof of the pulp chamber, significantly reduces the fracture resistance of endodontically treated teeth.^[4]^ As a result, timely restoration after RCT is crucial for protecting the tooth and preventing further damage.^[5]^

Previous studies have demonstrated that timely and appropriate restoration after RCT is critical for preserving tooth structure and maintaining its biomechanical properties. For instance, a study by Karamifar et al highlighted the importance of post-RCT restoration in preventing further tooth deterioration.^[1]^ Another study by Haridy et al showed that the biomechanical behavior of teeth can be significantly influenced by the amount of tooth structure loss and the type of restorative technique used.^[6]^ In addition to conventional mechanical and adhesive approaches, diode laser irradiation – particularly at 980 nm – has gained popularity because of its bactericidal effect, biostimulation capacity, and ability to decontaminate root-canal walls and periradicular tissues with minimal thermal side effects.^[7]^ However, there is limited consensus on the optimal restoration timing and method, particularly in terms of balancing fracture resistance and preservation of natural tooth structure.

The primary aim of this study was to evaluate the impact of different restoration timings on the fracture resistance of teeth after RCT. Additionally, we aimed to compare the effectiveness of 3 common restoration methods – full crown, onlay, and occlusal veneer – in enhancing fracture resistance. Understanding these factors can provide valuable guidance for clinicians in selecting appropriate restoration strategies post-RCT. Furthermore, this study seeks to address the challenges associated with preserving natural tooth structure while ensuring adequate mechanical strength and long-term functionality.

## 2. Methods

### 2.1. Study design

This prospective study aimed to evaluate the effect of different restoration timings following RCT on the fracture resistance of teeth. A total of 60 extracted human molars were subjected to standardized RCT from January 2023 to January 2024 and then randomly assigned to 3 main groups based on restoration timing: immediate (within 1 week after RCT), early (2–3 weeks after RCT), and delayed (4–6 weeks after RCT), with 20 teeth in each group. Furthermore, each main group was subdivided into 3 subgroups according to restoration method, namely full crown, onlay, and occlusal veneer, with 6 to 7 teeth in each subgroup. The study was conducted in accordance with the Declaration of Helsinki, and approval was obtained from the Institutional Review Board.

### 2.2. Study sample

A total of 60 extracted human molars were collected from patients undergoing orthodontic treatment. The inclusion criteria were as follows: teeth without caries, cracks, or previous restorations; teeth from patients without underlying medical conditions such as diabetes, hypertension, or renal insufficiency. The teeth were cleaned, disinfected, and stored in 0.1% thymol solution at 4°C until use.

### 2.3. Intervention methods

All teeth underwent standardized RCT by an experienced endodontist. The RCT procedure included access cavity preparation, root canal preparation, and root canal obturation. The restorative procedures were performed by an experienced restorative dentist as follows:

Full crown restoration, a full-coverage zirconia crown (KATANA™ Zirconia, Kuraray Noritake Dental Inc., Okayama, Japan) was fabricated for each tooth. The teeth were prepared with a 1.5 mm reduction on all surfaces, and impressions were taken using polyvinyl siloxane (Aquasil Ultra, Dentsply Sirona, York). The crowns were cemented using dual-cure resin cement (RelyX U200, 3M ESPE, St. Paul).

Onlay restoration, a ceramic onlay (IPS e.max CAD, Ivoclar Vivadent, Schaan, Liechtenstein) was fabricated to restore the occlusal and proximal surfaces. The teeth were prepared with a 1.2 mm reduction on the occlusal surface and 1.0 mm on the proximal surfaces. Impressions were taken, and the onlays were cemented using the same resin cement.

Occlusal veneer restoration, a composite occlusal veneer (Tetric PowerFill, Ivoclar Vivadent, Fürstentum, Liechtenstein) was applied directly to the occlusal surface. The surface was etched with 37% phosphoric acid (Condent Acid Etch Gel, Condent Dental Manufacturing Co, Schaan, Liechtenstein), bonded with a dentin bonding agent (AdheSE One, Ivoclar Vivadent), and the composite was light-cured for 20 seconds using a light-curing unit (Bluephase, Ivoclar Vivadent) with an intensity of 1200 mW/cm².

### 2.4. Data collection and outcomes

The fracture resistance of the teeth was tested using a universal testing machine (Instron 5966, Instron Corp., Norwood) at a crosshead speed of 1.0 mm/min. A steel ball (5 mm diameter) was used to apply a vertical load to the central fossa of the occlusal surface until fracture occurred. The fracture load was recorded in Newtons (N). The fracture mode was observed using a stereomicroscope (Leica MZ6, Leica Microsystems, Wetzlar, Germany) and classified as repairable (e.g., crack within the restoration or small separation between restoration and tooth) or non-repairable (e.g., tooth root fracture or large separation).

### 2.5. Statistical analysis

Statistical analysis was performed using SPSS software (version 26.0, IBM Corp, Armonk ). The fracture resistance data were expressed as mean ± standard deviation (SD). A one-way analysis of variance (ANOVA) was used to compare the mean fracture loads among the 3 restoration timing groups (immediate, early, and delayed). Post hoc Tukey tests were conducted to identify pairwise differences between groups. Within each restoration timing group, comparisons of fracture resistance among the 3 restoration methods (full crown, onlay, and occlusal veneer) were also analyzed using one-way ANOVA followed by post hoc Tukey tests. For the fracture mode analysis, the proportion of repairable and non-repairable fractures was compared across restoration timing groups using the Chi-square test. A significance level of *P* < .05 was used for all statistical tests.

## 3. Results

### 3.1. Sample distribution

The 60 teeth were randomly divided into 3 main groups based on restoration timing, with 20 teeth in each group. Each main group was further divided into 3 subgroups based on restoration method, with 6 to 7 teeth in each subgroup. The distribution of teeth across the groups is detailed in Table 1.

**Table 1:** Distribution of teeth across restoration timing and method groups.

Restoration timing	Subgroup	Number of teeth
Immediate restoration	Full crown	7
	Onlay	6
	Occlusal veneer	7
Early restoration	Full crown	7
	Onlay	6
	Occlusal veneer	7
Delayed restoration	Full crown	6
	Onlay	7
	Occlusal veneer	7

### 3.2. Fracture resistance

#### 3.2.1. Effect of different restoration timings on fracture resistance

The immediate restoration group exhibited the highest mean fracture load (2356.45 ± 412.68 N), followed by the early restoration group (2085.67 ± 389.45 N) and the delayed restoration group (1754.32 ± 356.78 N). A one-way ANOVA revealed a statistically significant difference in fracture resistance among the 3 groups (*P* < .05). post hoc Tukey tests showed that the immediate restoration group had significantly higher fracture resistance than the early and delayed groups (*P* < .05), and the early restoration group had higher fracture resistance than the delayed group (*P* < .05). The mean fracture loads for different restoration timings and their corresponding *P*-values are presented in Table 2.

**Table 2:** Effect of different restoration timings on fracture resistance.

Restoration timing	Mean fracture load (N)	*P*
Immediate (<1 wk)	2356.45 ± 412.68	.019
Early (2–3 wk)	2085.67 ± 389.45	.012
Delayed (4–6 wk)	1754.32 ± 356.78	–

#### 3.2.2. Effect of different restoration methods on fracture resistance

Within each restoration timing group, the full crown subgroup demonstrated significantly higher fracture resistance compared to the onlay and occlusal veneer subgroups (*P* < .05). However, no significant difference was observed between the onlay and occlusal veneer subgroups (*P* > .05). The mean fracture loads for each subgroup and their corresponding *P*-values are presented in Table 3.

**Table 3:** Effect of different restoration methods on fracture resistance.

Restoration timing	Restoration method	Mean fracture load (N)	*P*
Immediate	Full crown	2543.21 ± 389.56	–
	Onlay	2167.89 ± 321.45	.002
	Occlusal veneer	2089.34 ± 298.76	.003
Early	Full crown	2215.67 ± 356.78	–
	Onlay	1987.45 ± 312.67	.015
	Occlusal veneer	1895.67 ± 289.45	.021
Delayed	Full crown	1895.67 ± 345.67	–
	Onlay	1689.34 ± 298.76	.034
	Occlusal veneer	1621.56 ± 278.90	.042

### 3.3. Fracture mode

In the immediate restoration group, 85% of fractures were repairable, primarily manifested in the repair of cracks in the body or the separation of a small part of the prosthesis from the tooth. In the early restoration group, 65% of fractures were repairable, while in the delayed restoration group, only 45% of fractures were repairable. The proportion of non-repairable fractures, such as tooth root fracture, increased with delayed restoration timing (Table 4).

**Table 4:** Tooth fracture mode of different restoration times.

Restoration timing (n = 20)	Repairable (%)	Non-repairable (%)	*P*
Immediate	17 (44%)	3 (14%)	–
Early	13 (33%)	7 (33%)	.03
Delayed	9 (23%)	11 (52%)	.01

## 4. Discussion

The results of this study highlight the critical role of restoration timing in enhancing the fracture resistance and clinical prognosis of teeth after RCT. Earlier restoration (within 1 week) provided superior fracture resistance compared to delayed restoration, likely due to the timely protection of the remaining tooth structure and prevention of stress concentration and microcracks caused by occlusal forces.^[8]^ The loss of strength observed for each week of delay parallels the reduction reported by Kassis et al in molars left unrestored for 1 month.^[9]^ The study show that unstressed endodontic cavities accumulate hygroscopic microcracks, which defects coalesce under cyclic loading and explain the difference we measured between immediate and delayed groups. Early placement of an adhesive restoration seals dentinal tubules, halters water-gradient expansion and preserves the elastic modulus of residual crown tissue.^[10]^ Our findings align with these observations, as the delayed restoration group exhibited a higher proportion of non-repairable fractures and lower clinical success rates.

Among the restoration methods, full crown restoration consistently demonstrated significantly higher fracture resistance than onlay and occlusal veneer across all restoration timing groups, including immediate, early, and delayed restoration groups. This superior performance is attributed to the full coverage design of the crown, which distributes occlusal forces more evenly and provides greater structural support.^[11,12]^ However, the preparation of full crowns requires the removal of a significant amount of healthy tooth structure. In contrast, the onlay and occlusal veneer, while less invasive and preserving more natural tooth structure, also provided adequate fracture resistance, particularly in the immediate and early restoration groups. The success rates of onlay and occlusal veneer were comparable to those of full crowns in these groups, indicating their viability as alternatives when timely restoration is performed.^[13,14]^ It is important to note that the fracture resistance of onlay and occlusal veneer decreased significantly in the delayed restoration group. This may be related to further weakening of the tooth structure and uneven stress distribution between the restoration and the tooth. Therefore, when selecting onlay or occlusal veneer, it is crucial to ensure that restoration is performed early to maximize their benefits.^[15]^

The delayed restoration group exhibited a significantly higher proportion of non-repairable fractures. These fractures, typically characterized by root fractures or extensive tooth separation, often result in tooth loss and the need for extraction. This observation underscores the critical importance of timely restoration, even when minimally invasive methods are chosen.^[16]^ The increased incidence of non-repairable fractures in the delayed restoration group suggests that prolonged exposure to occlusal forces without protection leads to cumulative damage, ultimately resulting in more severe fractures.^[8,17]^ Research demonstrates that in long-term chewing simulations, dental tissues in delayed-repair scenarios show substantially more fatigue-damage accumulation. This accumulation gradually compromises the structural integrity of dental tissues, ultimately leading to irreparable-fracture occurrence.^[18]^ Future research could further investigate the specific mechanisms behind non-repairable fractures caused by delayed restoration, such as microcrack propagation, tooth structure fatigue, and periodontal ligament degeneration.

The findings of this study provide valuable guidance for clinical practice in post-root canal restoration strategies. First, early restoration (within 1 week) should be considered the standard approach for protecting teeth after RCT, particularly in high-risk patients (e.g., those with high occlusal forces). Second, while full crown restoration offers superior fracture resistance, its higher invasiveness may not be suitable for all patients. In cases where early restoration is ensured, onlay and occlusal veneer can serve as reasonable alternatives that balance fracture resistance and tooth structure preservation.

However, this study has certain limitations. The use of extracted teeth as samples does not fully replicate the complex clinical environment, which includes factors such as patient occlusal forces, oral hygiene, and periodontal health. Additionally, the study did not evaluate the long-term performance of different restoration materials, such as aging, fatigue effects, and bond stability with tooth structure. Future research could further explore these factors to better understand their impact on fracture resistance.

## 5. Conclusion

This study concludes that earlier restoration after RCT significantly enhances the fracture resistance of teeth and improves clinical prognosis. While full crown restoration offers superior protection, onlay and occlusal veneer are viable alternatives that balance fracture resistance and tooth structure preservation. Clinicians should prioritize timely restoration and consider the specific clinical situation and patient preferences when selecting the appropriate restoration method.

## Author contributions

**Conceptualization:** Yunyu Yuan, Pingping Chen.

**Data curation:** Yunyu Yuan, Pingping Chen.

**Formal analysis:** Yunyu Yuan.

**Investigation:** Pingping Chen.

**Methodology:** Pingping Chen.

**Project administration:** Yunyu Yuan.

**Software:** Yunyu Yuan.

**Validation:** Pingping Chen.

**Writing – original draft:** Yunyu Yuan, Pingping Chen.
